# Prevalence and Factors Associated with Substance Use and Misuse among Kosovar Adolescents; Cross Sectional Study of Scholastic, Familial-, and Sports-Related Factors of Influence

**DOI:** 10.3390/ijerph13050502

**Published:** 2016-05-16

**Authors:** Enver Tahiraj, Mladen Cubela, Ljerka Ostojic, Jelena Rodek, Natasa Zenic, Damir Sekulic, Blaz Lesnik

**Affiliations:** 1 Ministry of Culture Youth and Sport, Pristina 10000, Kosovo; envertahiraj@live.com; 2 College Universi Bardhosh, Pristina 10000, Kosovo; 3 Faculty of Medicine, University of Mostar, Mostar 88000, Bosnia and Herzegovina; mladen.cubela@gmail.com (M.C.); ljerka.ostojic@sve-mo.ba (L.O.); 4 Faculty of Kinesiology, University of Split, Split 21000, Croatia; jelena.rodek@kifst.hr (J.R.); natasazenic@yahoo.com (N.Z.); 5 Academy of Medical Sciences, Sarajevo 88000, Bosnia and Herzegovina; 6 Department of Health Care Studies, University of Split, Split 21000, Croatia; 7 Faculty of Sport, University of Ljubljana, Ljubljana 1000, Slovenia; blaz.lesnik@fsp.uni-lj.si

**Keywords:** substance abuse, association, sport, education

## Abstract

Adolescence is considered to be the most important period for the prevention of substance use and misuse (SUM). The aim of this study was to investigate the problem of SUM and to establish potentially important factors associated with SUM in Kosovar adolescents. Multi-stage simple random sampling was used to select participants. At the end of their high school education, 980 adolescents (623 females) ages 17 to 19 years old were enrolled in the study. The prevalence of smoking, alcohol consumption (measured by Alcohol Use Disorder Identification Test–AUDIT), and illegal drug use (dependent variables), as well as socio-demographic, scholastic, familial, and sports-related factors (independent variables), were assessed. Boys smoke cigarettes more often than girls with daily-smoking prevalence of 16% among boys and 9% among girls (OR = 1.85, 95% = CI 1.25–2.75). The prevalence of harmful drinking (*i.e.*, AUDIT scores of >10) is found to be alarming (41% and 37% for boys and girls, respectively; OR = 1.13, 95% CI = 0.87–1.48), while 17% of boys and 9% of girls used illegal drugs (OR = 2.01, 95% CI = 1.35–2.95). The behavioral grade (observed as: excellent–average-poor) is the factor that was most significantly correlated with SUM both in boys and girls, with lower behavioral grades among those adolescents who consume substances. In girls, lower maternal education levels were associated with a decreased likelihood of SUM, whereas sports achievement was negatively associated with risky drinking. In boys, sports achievement decreased the likelihood of daily smoking. Information on the factors associated with SUM should be disseminated among sports and school authorities.

## 1. Introduction

Substance use and misuse (SUM), which includes cigarette smoking, alcohol consumption, illegal drug use (*i.e.*, opiates, cannabinoids, sedatives and other prescription drugs), as well as other behaviors, is one of the leading public-health problems in the world today [1,2,3]. Although it is decreasing globally, tobacco smoking remains the leading cause of premature death; combined with obesity, smoking is the leading cause of healthcare costs [1,4,5]. In addition to being serious health-threatening behaviors, alcohol consumption and illegal drug use are associated with numerous other problems such as trauma, traffic accidents, crime, aggression, violence, *etc.* [6,7,8].

Adolescence is considered to be the most important period for the prevention of SUM [1,9]. Evidence suggests that individuals who do not consume these substances before the age of 21 are less likely to consume them later in life [10]. Therefore, special efforts are needed to develop effective public health policies for the prevention of substance misuse in adolescents. Determining social and cultural-specific factors associated with substance misuse in adolescents is particularly important [11].

Kosovo is a country located on the Balkan Peninsula, in Southeastern Europe, which is inhabited mostly by native Albanians. After recent wars, Kosovo was the last part of former Yugoslavia to declare formal political independence. Although statistics on SUM are not systematic, several studies have reported a high prevalence of SUM in adolescents [12,13,14]. Although this prevalence may be partly due to the influence of the social and cultural acceptance of cigarette smoking and alcohol consumption, substance availability, post-war trauma and ineffective public health policy against SUM, especially for adolescents, may also be contributing factors.

The factors associated with SUM in adolescents have been frequently investigated [1,10,15]. However, it is generally accepted that gender-specific and culturally specific approaches are required for studying the problem of SUM and its correlates in different countries. The factors associated with SUM in adolescence are rarely “general”; the factors found to be significantly associated with SUM in one socio-cultural environment may be insignificant or even inversely correlated with SUM in another setting (*i.e.*, community, gender, ethnicity) [11]. Previous studies conducted in the region of former Yugoslavia identified familial variables, sport-related factors and scholastic variables as being associated with SUM in adolescents [1,10,16]. However, there is no study that specifically investigated the prevalence of the use and misuse of different substances and the factors associated with SUM in Kosovar adolescents.

The aim of this study was to report the prevalence of SUM and to identify potentially important factors associated with SUM in Kosovar adolescents. Specifically, we examined daily consumption of cigarettes, risky consumption of alcohol, use of other drugs, and their association with familial, scholastic, and sports-related factors.

## 2. Materials and Methods

### 2.1. Subjects

**Figure 1:**
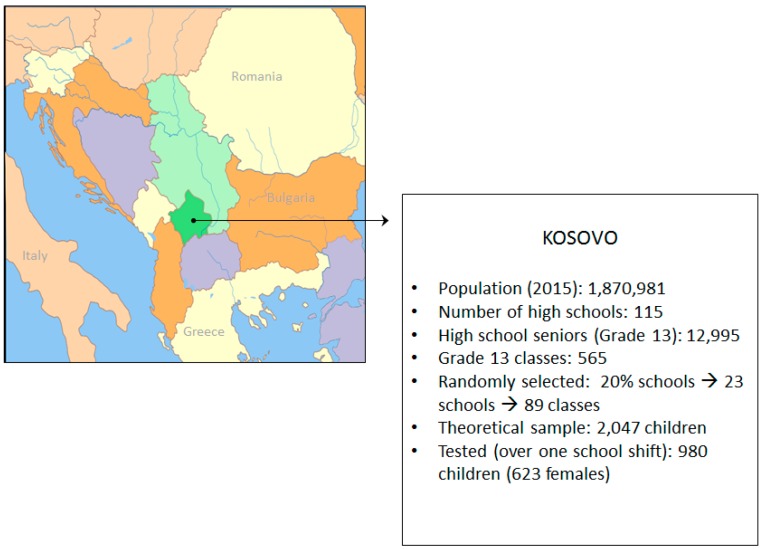
Geographical location of Kosovo and sampling procedure.

This study enrolled 980 adolescents (623 females) aged 17 to 19 years old at the end of their high school education (13th grade). The sample was intentionally selected because previous studies conducted in the territory of former Yugoslavia enrolled subjects of the same age, which allowed for a meaningful comparison of the results. The sample was tested during the 2014/2015 school year and represents approximately 7.5% of high school seniors in Kosovo. The sampling method is presented in Figure 1.

Multi-stage simple random sampling was used to select participants. First, we selected one-fifth of the high schools in the territory of Kosovo by lottery, which resulted in 85 final-year classes and a theoretical sample size of 2047 children. Finally, all high school seniors present on the day of the survey were invited to participate. Consequently, the sample included 980 participants (approximately one-half of the theoretical sample). Although the sampling procedure involved the random sampling of high schools, the final sample included more females than males mostly because of the difference between boys and girls in the 12th school grade (*i.e.*, level necessary for a continuation at college/university education).

### 2.2. Variables

In this study, we administered a questionnaire that had been previously used and validated in the region [1,11]. The inventory consisted of questions asking subjects about SUM (consumption of cigarettes, alcohol, and other drugs), scholastic, sports-related and socio-demographic (*i.e.*, familial) factors.

Socio-demographic data included age, gender and familial variables, including (i) maternal and (ii) paternal educational level (using a three-point scale including “elementary school”, “high-school”, and “college-university degree”) and (iii) self-estimated socio-economic status (SES; three-point scale: “below-average”, “average” and “above-average”).

To enable meaningful comparisons with results presented previously for the territory of former Yugoslavia, [1,10] sports-related factors were evaluated using two questions: (i) the amount of time subjects spent engaging in sports (using a four-point scale “Never involved in sports”, “Less than a year”, “1–5 years”, and “5+ years”); and (ii) subject’s competitive sports achievements (using a three-point scale including “Never competed”, “Lower ranks” and “National level competitions and higher”). Scholastic factors were assessed by reporting (i) grade point average (four point scale including “Excellent/Very good”, “Average”, “Below-average and failed”) and (ii) behavioral grade (three point scale including “Excellent”, “Average” and “Poor”) from the preceding semester.

Cigarette smoking was assessed on a six-point scale (“Never smoked”, “Quit”, “Cigarette or two from time to time, but not daily”, “Daily”, “More than 10 cigarettes”, and “More than a pack daily”). The subjects were later grouped as “daily-smokers” (last three answers) and “non-smokers” (those who answered Never smoked, Quit, or A cigarette or two from time to time but not daily). The purpose of this investigation was to study factors associated with smoking and not “experimentation with cigarettes”. Although a characteristic of the cultural heritage in the region often includes smoking a cigarette or two during special occasions (familial gatherings, festivals, *etc.*), we decided to code “daily smoking” as a categorical variable.

Alcohol consumption was measured using the Alcohol Use Disorder Identification Test (AUDIT) questionnaire, which contains 10 items with scores ranging from 0 to 4 for a hypothetical minimum (0) to maximum (40) range. The results were later divided into “harmful (risky) drinking” (scores of 11 or above) and “non-harmful (non-risky) drinking” (scores below 11) [11]. The scale for consumption of other drugs consisted of questions about the consumption of marijuana, hashish, heroin, cocaine, party drugs (e.g., ecstasy, amphetamines, and others), inhalants, and sedatives. A six-point range of consumption was offered for each question (”Never”, “Ever tried”, “Once or twice”, “3–5 times”, “6–9 times” and “10 times or more”). For the purpose of logistic regression analysis, subjects were categorized as “non-users” and “users” (subjects who declared that they had used as least one substance more than twice).

### 2.3. Testing and Ethics

The survey was conducted in a single day. All sampled adolescents who were at school on that day were included in the study. Throughout mandatory school meeting, which was held a week before the study, at least one parent of each child was introduced to complete procedure and study aims, and provided a written consent for his/hers child participation. Subjects who were at least 18 years old provided their own written consent for participation. All of the survey questions had multiple-choice answer options. The testing groups consisted of at least 10 adolescents. Each respondent was informed that the survey was strictly anonymous, that they could refuse to participate, and that they could leave questions and/or the entire questionnaire unanswered. Each respondent received the questionnaire and one envelope. When the survey was completed, the subject placed the questionnaire in the envelope, sealed it and deposited it in a closed box. The box was not opened until the next day to ensure the anonymity of the testing. The study was approved by the Institutional Ethical Board of the Faculty of Kinesiology, University of Split (Approval No.: 2181-205-02-05-14-005; 11 September 2014).

### 2.4. Statistics

The descriptive statistics included counts (frequencies) and percentages, as well as means ± standard deviations. Differences between genders were assessed using Kruskal Wallis ANOVA or t-tests, depending on the parametric/non-parametric nature of the variables. The prevalence of (i) daily smoking; (ii) risky drinking and (iii) consumption of other drugs was compared between genders by calculating the Odds Ratio (OR) with the 95% Confidence Interval (95% CI).

The associations between covariates and the types of substance misuse were analyzed using multivariate logistic regressions. Multiple regression approach allowed us to control for possible interactions between covariates. The binomial logistic regression criteria were: (i) daily cigarette smoking (no-yes); (ii) risky alcohol consumption (no-yes); and (iii) illegal drug consumption (no-yes). For each criterion (smoking, drinking and drug use), we calculated three multiple regression models. The predictors were: (a) scholastic factors (grade point average and behavioral grade); (b) sports-related factors (time of sport involvement and sport achievement); and (c) familial variables (paternal education, maternal education, and SES). In all logistic regression models, subject’s age was included as a confounding factor. Since previous studies identified variable covariates between genders, the logistic regressions were stratified by gender [1,13,14].

## 3. Results

The scholastic factors, sport factors and familial variables are presented in the Supplementary material (Table S1). The reported use of cigarettes, (53% and 67% non-smokers for boys and girls, respectively) cannabis (88% and 97% non-users for boys and girls, respectively) and hashish (94% and 99% non-users for boys and girls, respectively) was higher among boys, but girls used sedatives more often than boys (94% and 88% non-smokers for boys and girls, respectively) (Supplementary material; Table S2).

**Figure 2:**
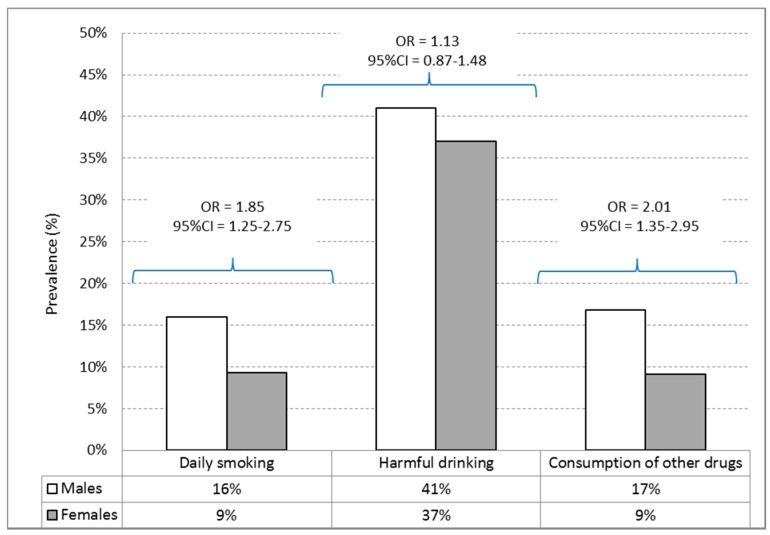
The prevalence of daily cigarette smoking, risky drinking and use of other drugs with odds ratios (OR) and 95% confidence intervals (95% CI) between genders. The reported frequency percentages are based on a sample size of 357 for males and *n* = 623 for females.

Daily smoking was more prevalent among boys (16%) than among girls (9.3%; OR = 1.85; 95% CI = 1.25–2.75). The prevalence of consumption of illegal drugs was higher among boys (16.8%) than among girls (9.15%; OR = 2.01; 95% CI = 1.35–2.95). There was no significant difference in the prevalence of harmful alcohol consumption between boys (40.6%) and girls (37.4%; OR = 1.13; 95% CI = 0.87–1.48) (Figure 2).

The logistic regression analyses indicated a lower behavioral grade (for boys and girls) and lower grade-point-average (for girls) for participants who consumed cigarettes on a daily basis. Sports-related factors were significantly (*p* < 0.05) associated with daily cigarette smoking only in boys; the highest level of sports achievement was associated with a decreased likelihood of daily smoking. For boys, lower maternal education was associated with an increased likelihood of daily smoking. For girls, the likelihood of daily smoking decreased with lower maternal education (Table 1). For boys and girls, risky alcohol consumption was more prevalent in those who had earned average behavioral grades. Among girls, the likelihood of risky alcohol consumption increased with sports achievement, and decreased with lower maternal education (Table 2).

Among boys, the behavioral grade was correlated with drug use. Sports-related factors were not associated with drug use. Maternal education was correlated with consumption of illegal drugs in girls (Table 3).

## 4. Discussion

This study had several important findings. First, the observed predictors were specifically related to SUM variables in Kosovar boys and girls. In general, scholastic variables were the most significant predictors of SUM in boys and girls. Among boys, the observed SUM variables were differentially associated with scholastic, familial and sports-related factors. Among girls, maternal education and sport participation were factors that increased the risk of SUM. The background for these results will be addressed following the discussion of the prevalence of observed SUM in boys and girls.

### 4.1. The High Prevalence of Tobacco Smoking and Alcohol Drinking

This study confirmed previous reports of a high prevalence of SUM in adolescents from former Yugoslavian territories [10,11,13]. Therefore, our findings of a smoking prevalence of 41% among boys and 31% among girls (with 16% and 9%, respectively, being daily smokers) falls within an expected range. When compared to European countries, where the highest prevalence of smoking has been reported to be in Hungary and Belgium (17%–19%), and Austria (20%–25%), the data from Kosovo are clearly alarming [17]. There are several explanations for such disturbing findings.

One of the factors known to be directly associated with the prevalence of smoking is the price of tobacco products, which is mostly related to tobacco taxes; these taxes are an essential component of a comprehensive tobacco control strategy [18,19]. The price of tobacco products in the former Yugoslavian territories is relatively low. The price of a pack of cigarettes rarely exceeds 3 USD, which is almost 3–6 times lower than the price of tobacco products in EU countries [16]. Thus, the low price of tobacco almost certainly directly contributes to smoking patterns. Another factor is that smoking is socially accepted and there are no strict regulations against selling tobacco products to minors.

The situation for alcohol price is similar to that for cigarettes. For example, a bottle of spirits costs up to 10 USD, which is at least 2–3 times cheaper than the price in European countries. Native Albanians (the majority of Kosovar residents) are Muslims, though this does not appear to be a factor in the lower levels of alcohol consumption. In a recent study that examined SUM in adolescent Muslims from other parts of former Yugoslavia, Bosnia and Herzegovina, the authors reported a relatively high prevalence of alcohol consumption, regardless of the religious affiliation of the participants [16].

The prevalence of harmful alcohol consumption among adolescents in this study is similar to that previously reported in the region [10,11]. However, the misuse of alcohol goes beyond “statistics.” The similar prevalence of alcohol consumption in boys and girls is already reported and requires special attention [14]. Alcohol consumption is known to be directly associated with delinquency, transactional sex, prostitution, and even sex trafficking. According to UNICEF warnings (cited), “Kosovo is currently a place of origin, destination and internal trafficking in girls and women for sexual exploitation. Whereas the existence of trafficking into Kosovo of foreign women and girls has been known since 1999, the existence of trafficking in Kosovar women and girls has only recently been fully acknowledged” (end of citation) [20]. The high prevalence of alcohol consumption in females is therefore particularly alarming.

The consumption of drugs is not alarming. Figures reported herein for Kosovar adolescents are similar to those previously reported for other parts of the former Bosnia and Herzegovina and Croatia [1,10,16]. Also, the differences between genders are also as expected, with only minority of adolescents that consume marihuana/hashish (boys), and sedatives (girls) [11].

### 4.2. Scholastic Variables and SUM

Scholastic achievement is frequently reported to be negatively associated with substance misuse in adolescence [10,21]. Regardless of the type of scholastic achievement (grade point average, drop-out from school, absence from school, *etc.*), children who consume alcohol, smoke cigarettes, and consume illegal drugs are more likely to perform poorly in school [1]. We noted a stronger association between the behavioral grade (as a measure of scholastic achievement) and SUM than between grade point average and SUM. To explain these findings, a brief overview of proposed theories on the negative association between scholastic achievement and SUM in adolescence is needed.

Three theories are primarily used to describe the association between SUM and academic failure. According to one theory, cigarette smoking is thought to impair learning capacity because of the known (physiological) negative effects of smoking on cognitive function [22,23]. This theory has been criticized because of the time lag required for the negative effects of smoking on learning to manifest, and therefore such effects are not likely in adolescence [11,16]. The second theory brings the socio-cultural context of SUM into focus. Children who fail academically are frequently absent from school (*i.e.*, they are avoiding classes), which places them in socio-cultural situations in which they are more likely to initiate SUM. According to this theory, academic failure is therefore “the cause” of SUM [16,21]. However, our results actually support the third explanation for the negative association between scholastic achievement and SUM in adolescence.

The third explanation is based on the “theory of problem behavior”, which centers on the notion of a psychosocial tendency for unconventionality [24]. According to this theory “problem behaviors” often appear in “tandem” primarily because certain people have a “general tendency” for such behaviors. Therefore, those adolescents who are leaning toward SUM are often “unconventional.” The behavioral grade in the Kosovar educational system is not defined by purely objective indicators but largely depends on the instructor’s (subjective) opinion of each student. Therefore, it is not uncommon for “unconventionality” to result in a poor behavioral grade but not necessarily in a low grade point average (*i.e.*, grade point average is a more “objective” indicator of a student’s achievement level).

### 4.3. Sports-Related Factors and SUM

Sports provide numerous benefits to young people [25]. In addition to the known health-related benefits of sports, such as improved cardiovascular endurance, muscular fitness, and the prevention of obesity, sports are frequently considered to be a mechanism of encouraging pro-social behavior [26]. As a result, active participation in sports is often considered to be a protective factor against SUM in adolescence. However, studies conducted thus far have not consistently confirmed that SUM is less prevalent in athletic children than in non-athletic children [1,11,27]. Therefore, our findings of a varying association between SUM and sports-related factors are not surprising.

Despite the known negative effects of alcohol consumption on physical performance (*i.e.*, dehydration, altered recovery, risk of injury, *etc.*), alcohol consumption in sports is a known problem [28]. An increased prevalence of risky alcohol consumption and alcohol-related harm has been reported in members of sports groups compared with non-sports populations [29]. Therefore, the association between sports achievement and alcohol consumption (*i.e.*, girls who achieved higher competitive results in sports were more likely to be engaged in harmful drinking patterns) is not unexpected. The association between higher alcohol consumption and high levels of achievement in sports is almost certainly a consequence of: (i) post-game gatherings occurring after (successful) competitions; and (ii) frequent out of home situations for girls competing at higher levels. Both circumstances increase the likelihood of alcohol consumption.

Among boys, sports achievement was protective against smoking. The analysis identified boys who achieved the highest results (*i.e.*, competed and succeeded at the highest level) as having the lowest risk for smoking. Because of the known negative influence of smoking on physical capacity, the negative association between sports achievement and smoking is logical [30].

### 4.4. Familial Variables and SUM

The findings of the studies examining parental education levels as a factor that influences SUM in children are not consistent. In some cases, investigators reported lower SUM in adolescents whose parents were better educated [31,32]. However, for each investigation that reported such an association, another reported an opposite association [33,34]. In a recent study that separately investigated the education level of both parents, the authors reported that the mother’s education level was inversely associated with youth SUM, whereas the father’s education level was positively correlated with alcohol use among girls [15]. Therefore, our results regarding gender-specific variable associations between parental education and SUM are not surprising.

A higher prevalence of smoking, alcohol consumption and illegal drug use was found among girls whose mothers were better educated. Compared to other parts of former Yugoslavia, Kosovo is known to be a country where traditionalism is highly prevalent [35]. This characteristic resulted in vast differences between the education levels of males and females, which are also mirrored in our results (42% and 27% with college/university-level education for fathers and mothers, respectively).

Previous studies have noted that societies with traditionally restrictive gender roles do not accept SUM among women. There is also the concern that women may lean more toward SUM if rapid social change leads to an alteration in traditional gender norms that discourage such behaviors [36]. As a result, we believe that it is not the case that higher maternal education level “per-se” increases SUM in girls, but rather that those girls whose mothers are better educated come from families that are more oriented toward non-traditional roles of women, simultaneously resulting in higher maternal education and SUM levels among girls.

Although frequently studied, we did not find that socioeconomic status (SES) was systematically associated with SUM in adolescents. Some researchers have reported a higher prevalence of SUM in low-SES children, whereas others found a decreased likelihood of SUM in children of lower SES [37,38,39]. Additionally, in some cases, researchers reported gender-specific or racial/ethnic associations between SES and SUM [13,40]. We found no significant association between SES and SUM, which may have been because we studied adolescents from different parts of Kosovo.

### 4.5. Limitations

The main limitation of the study is that the data were self-reported. However, we believe that the strict anonymity of the testing and the fact that we tested the children at the end of their mandatory education decreased the possibility that they did not respond honestly. The study was retrospective, and consequently, we cannot determine with certainty a cause-effect relationship between the studied factors. Additionally, we must note that Kosovo is a country where both smoking and alcohol consumption are socially acceptable. Therefore, the generalizability of the findings is limited to similar socio-cultural environments. With regard to observed sport-covariates, we must acknowledge that in this study we hadn’t observed the intensity of sport-involvement (*i.e.* training sessions per week, intensity of exercise). Therefore, in future studies these issues should be more precisely addressed. Additionally, in this study only cigarette smoking was assessed, but there is increasing evidence of the prevalence of other types of smoking. Finally, as a certain methodological remark, we must note that testing on AUDIT scale generally allows an interpretation of three separate sub-scales (*i.e.*, “hazardous alcohol use”, “dependence symptoms” and “harmful alcohol use”). However, in this study we were focused only at “harmful drinking” which is evidenced as a sum of all three sub-scales. While in this study we have observed other substances also, the additional analyses of the AUDIT sub-scores will broaden the discussion out of this paper’s primary scope (*i.e.*, studying prevalence and covariates for different types of SUM). Nonetheless, given the lack of studies evaluating the factors associated with SUM in Kosovar adolescents and the high prevalence of SUM in this country, we believe that this study expands our knowledge in this field.

## 5. Conclusions

Effective public health programs to prevent SUM in adolescents should be based on the socio-cultural characteristics and factors that influence SUM. This study identified several important factors associated with SUM in Kosovar adolescents. Scholastic variables were systematically associated with SUM in both boys and girls. However, from our perspective, data on such associations should not be incorporated into public health interventions aimed at preventing SUM. Although the association between scholastic variables and SUM is primarily related to a (relatively) lower behavioral grade in those children who misuse substances, we consider this relationship to be mostly modulated by the unconventionality of those children who are prone to substance abuse. Girls who achieve higher competitive sports results are more likely to engage in risky alcohol consumption. This information should be widely and systematically disseminated to sports authorities (sport clubs, sport organizations, *etc.*) because of their direct involvement in organizing post-game gatherings where alcohol is consumed. Because the data on harmful alcohol consumption indicate a high prevalence of such behavior in girls, urgent action is required. Parents should be informed of the systematic association between higher maternal education and an increased prevalence of SUM in girls. This information could be discretely disseminated as a part of regular school administrative functions. For example, regular teacher-parent meetings might be an appropriate way for parents to learn about these issues. The information should be presented in such a way that parents are directly informed of a (potential) problem, while not being labeled as “responsible.” These meetings would likely be more effective than one-on-one discussions.

## Figures and Tables

**Table 1:** Logistic regression models for daily smoking in boys and girls.

	Boys (*n* = 357)	Girls (*n* = 623)
	OR (95% CI)	OR (95% CI)
**Model 1: Scholastic Factors**		
*Grade Point Average*	*p* > 0.05	*p* < 0.05
Excellent/Very good	REF	REF
Average	0.03 (0.00–14.10)	4.24 (1.88–9.31)
Under-average and failed	1.65 (0.66–3.12)	5.89 (2.15–11.13)
*Behavioral Grade*	*p* < 0.05	*p* < 0.05
Excellent	REF	REF
Average	16.51 (2.62–23.63)	3.35 (1.63–6.86)
Poor	3.92 (2.07–7.41)	2.27 (0.55–9.32)
**Model 2: Sport Factors**		
*Time of The Involvement in Sports*	*p* > 0.05	*p* > 0.05
Never involved	REF	REF
Less than a year	0.60 (0.23–1.55)	1.33 (0.73–2.44)
1–5 years	1.22 (0.52–2.85)	0.60 (0.24–1.54)
More than 5 years	0.50 (0.18–1.34)	0.97 (0.32–2.98)
*Sport Competitive Achievement*	*p* < 0.05	*p* > 0.05
Never competed	REF	REF
Lower ranks	0.61 (0.26–1.42)	0.60 (0.21–1.76)
National level competitions and higher	0.35 (0.11–0.86)	0.01 (0.00–12.35)
**Model 3: Familial Factors**		
*Socio Economic Status*	*p* > 0.05	*p* > 0.05
Under average	1.58 (0.19–13.51)	0.12 (0.01–12.44)
Average	1.10 (0.49–2.51)	0.66 (0.03–45.96)
Above average	REF	REF
*Maternal Education*	*p* < 0.05	*p* < 0.05
Elementary school	2.11 (0.76–5.11)	0.07 (0.09–0.32)
High school	0.31 (0.16–0.89)	0.29 (0.11–0.71)
College/University degree	REF	REF
*Paternal Education*	*p* > 0.05	*p* > 0.05
Elementary school	0.81 (0.09–6.55)	1.56 (0.45–15.88)
High school	0.68 (0.21–1.45)	2.11 (0.98–5.12)
College/University degree	REF	REF

The variables for each model were included simultaneously and are therefore adjusted for each other. The models were also adjusted for age.

**Table 2:** Logistic regression models for harmful drinking in boys and girls.

	Boys (*n* = 357)	Girls (*n* = 623)
	OR (95% CI)	OR (95% CI)
**Model 1: Scholastic Factors**		
*Grade Point Average*	*p* > 0.05	*p* > 0.05
Excellent/Very good	REF	REF
Average	0.90 (0.40–1.51)	2.01 (0.11–2.44)
Under-average and failed	1.71 (0.29–11.21)	6.11 (0.92–17.64)
*Behavioral Grade*	*p* < 0.05	*p* < 0.05
Excellent	REF	REF
Average	1.91 (1.16–3.15)	1.34 (1.13–2.19)
Poor	4.03 (0.68–23.88)	0.32 (0.09–1.09)
**Model 2: Sport Factors**		
*Time of the Involvement in Sports*	*p* > 0.05	*p* > 0.05
Never involved	REF	REF
Less than a year	0.74 (0.38–1.46)	1.02 (0.69–1.49)
1–5 years	1.63 (0.85–3.11)	1.27 (0.79–2.02)
More than 5 years	1.17 (0.59–2.30)	0.65 (0.33–1.29)
*Sport Competitive Achievement*	*p* > 0.05	*p* < 0.05
Never competed	REF	REF
Lower ranks	1.09 (0.64–1.85)	1.76 (0.98–3.06)
National level competitions and higher	1.22 (0.68–1.86)	2.85 (1.01–8.21)
**Model 3: Familial Factors**		
*Socio Economic Status*	*p* > 0.05	*p* > 0.05
Under average	0.21 (0.11–2.66)	0.66 (0.03–13.11)
Average	1.53 (0.87–2.74)	1.76 (0.34–34.51)
Above average	REF	REF
*Maternal Education*	*p* > 0.05	*p* < 0.05
Elementary school	1.27 (0.62–3.01)	0.11 (0.09–0.29)
High school	0.63 (0.34–1.19)	0.30 (0.23–0.51)
College/University degree	REF	REF
*Paternal Education*	*p* > 0.05	*p* > 0.05
Elementary school	0.44 (0.08–3.13)	2.00 (0.59–4.11)
High school	0.78 (0.21–1.11)	0.89 (0.62–1.34)
College/University degree	REF	REF

The variables for each model were included simultaneously and are therefore adjusted for each other. The models were also adjusted for age.

**Table 3:** Logistic regression models for consumption of illegal drugs in boys and girls.

	Boys (*n* = 357)	Girls (*n* = 623)
	OR (95% CI)	OR (95% CI)
**Model 1: Scholastic Factors**		
*Grade Point Average*	*p* > 0.05	*p* > 0.05
Excellent/Very good	REF	REF
Average	1.65 (0.71–3.11)	1.56 (0.79–2.32)
Under-average and failed	0.01 (0.00–5.61)	0.99 (0.52–2.54)
*Behavioral Grade*	*p* < 0.05	*p* > 0.05
Excellent	REF	REF
Average	2.37 (1.29–4.29)	1.53 (0.73–3.40)
Poor	10.53 (1.71–64.81)	3.29 (0.94–11.53)
**Model 2: Sport Factors**		
*Time of the Involvement in Sports*	*p* > 0.05	*p* > 0.05
Never involved	REF	REF
Less than a year	1.54 (0.67–3.56)	1.24 (0.66–2.34)
1–5 years	1.13 (0.49–2.63)	0.80 (0.34–1.89)
More than 5 years	0.65 (0.25–1.67)	1.51 (0.57–4.01)
*Sport Competitive Achievement*	*p* > 0.05	*p* > 0.05
Never competed	REF	REF
Lower ranks	0.65 (0.30–1.39)	1.32 (0.59–2.99)
National level competitions and higher	1.45 (0.69–3.03)	0.01 (0.00–111.98)
**Model 3: Familial Factors**		
*Socio Economic Status*	*p* > 0.05	*p* > 0.05
Under average	0.02 (0.01–34–11)	0.05 (0.13–32.556)
Average	3.60 (0.76–9.53)	1.01 (0.54–54.11)
Above average	REF	REF
*Maternal Education*	*p* > 0.05	*p* < 0.05
Elementary school	0.76 (0.45–1.91)	0.61 (0.26–1.11)
High school	0.97 (0.76–1.99)	0.54 (0.25–0.69)
College/University degree	REF	REF
*Paternal Education*	*p* > 0.05	*p* > 0.05
Elementary school	0.04 (0.00–4.12)	3.04 (0.57–5.93)
High school	0.66 (0.41–1.76)	1.44 (0.67–2.85)
College/University degree	REF	REF

The variables for each model were included simultaneously and are therefore adjusted for each other. The models were also adjusted for age.

## Supplementary Materials

The following are available online at  www.mdpi.com/1660-4601/13/5/502/s1, Table S1: Decriptive data (frequencies—f, and percentages—%) for the studied scholastic-, sport, and familial-factors, Table S2: Decriptive data (frequencies—f, and percentages—%) for cigarette smoking and consumption of other drugs, Differences between genders (Kruskal Wallis test H, level of significance—p).

## Abbreviations

The following abbreviations are used in this manuscript:

AUDIT	Alcohol Use Disorders Identification Test
SUM	substance use and misuse
SES	socio economic status
