# Supplementary material for: Prevalence and Factors Associated with Substance Use and Misuse among Kosovar Adolescents; Cross Sectional Study of Scholastic, Familial-, and Sports-Related Factors of Influence
Source: Int J Environ Res Public Health. 2016 May 16;13(5):502. doi: 10.3390/ijerph13050502 (PMC4881127; doi:10.3390/ijerph13050502)
Supplement: Supplementary file 1 [file ijerph-13-00502-s001.pdf]

Supplementary Materials: Prevalence and Factors Associated with Substance Use and Misuse among Kosovar Adolescents; Cross Sectional Study of Scholastic, Familial-, and Sports-Related Factors of Influence

Enver Tahiraj, Mladen Cubela, Ljerka Ostojic, Jelena Rodek, Natasa Zenic, Damir Sekulic and Blaz Lesnik

Table S1. Decriptive data (frequencies–f, and percentages—%) for the studied scholastic-, sport, and familial-factors.

	Total	Boys	Girls
	(*N* = 980)	(*N* = 357)	(*N* = 623)
	f (%)	f (%)	f (%)
**Grade Point Average**			
Excellent/very good	780 (79.67)	269 (75.4)	511 (82.0)
Average	174 (17.8)	82 (23)	92 (14.8)
Under-average and failed	26 (2.7)	6 (1.7)	20 (3.2)
**Behavioral Grade**			
Excellent	762 (77.8)	254 (71.1)	508 (81.5)
Average	192 (19.6)	95 (26.6)	97 (15.6)
Poor	26 (2.7)	8 (2.2)	18 (2.9)
**Time of the Involvement in Sport**			
Never involved	292 (29.8)	50 (14)	242 (38.8)
Less than a year	294 (30)	84 (23.5)	210 (33.7)
1–5 years	235 (24)	119 (33.3)	116 (18.6)
More than 5 years	159 (16.2)	104 (29.2)	55 (8.8)
**Sport Achievement**			
Never competed	738 (75.3)	205 (57.4)	533 (85.6)
Competitions of the lower ranks	146 (14.9)	77 (21.6)	69 (11.1)
National level competitions and higher	96 (9.8)	75 (21)	21 (3.4)
**Socio Economic Status (Family Finances)**			
Under Average	16 (1.6)	8 (2.2)	8 (1.3)
Average	809 (82.6)	291 (81.5)	518 (83.1)
Above average	155 (15.8)	58 (16.2)	97 (15.6)
**Paternal Education**			
Elementary school	18 (1.8)	6 (1.7)	12 (1.9)
High school	552 (56.3)	182 (50.9)	370 (59.4)
College/University degree	410 (41.8)	169 (47.3)	241 (38.7)
**Maternal Education**			
Elementary school	122 (12.4)	34 (9.5)	88 (14.1)
High school	592 (60.4)	213 (59.7)	379 (60.9)
College/University degree	266 (27.1)	110 (30.8)	156 (25)

Table S2. Decriptive data (frequencies–f, and percentages-%) for cigarette smoking and consumption of other drugs, Differences between genders (Kruskal Wallis test H, level of significance–p).

	Total	Males	Females	KW	
	(*N* = 980)	(*N* = 357)	(*N* = 623)		
	f (%)	f (%)	f (%)	H	*p*
Cigarette Smoking				18.14	0.01
Never smoked	605 (61.7)	189 (52.9)	416 (66.8)		
Quit	28 (2.9)	18 (5)	10 (1.6)		
From time to time. but not daily	232 (23.7)	93 (26.1)	139 (22.3)		
Daily. less than 10 cigarettes	37 (3.8)	14 (3.9)	23 (3.7)		
A pack daily	32 (3.3)	21 (5.9)	11 (1.8)		
More than a pack daily	46 (4.7)	22 (6.2)	24 (3.9)		
Ephedrine				3.75	0.06
Never	961 (98.1)	346 (96.9)	615 (98.7)		
Ever tried	10 (1)	8 (2.2)	2 (0.3)		
Once or twice	9 (0.9)	3 (0.8)	6 (1)		
Cocaine				3.53	0.06
Never	969 (98.9)	350 (98)	619 (99.4)		
Ever tried	6 (0.6)	4 (1.1)	2 (0.3)		
Once or twice	5 (0.5)	3 (0.8)	2 (0.3)		
SPEED				7.18	0.06
Never	969 (98.9)	348 (97.5)	621 (99.7)		
Ever tried	4 (0.4)	4 (1.1)	0 (0)		
Once or twice	2 (0.2)	2 (0.6)	0 (0)		
3–5 times	3 (0.3)	3 (0.8)	0 (0)		
6–9 times	2 (0.2)	0 (0)	2 (0.3)		
Ecstasy				3.53	0.06
Never	969 (98.9)	350 (98)	619 (99.4)		
Ever tried	6 (0.6)	4 (1.1)	2 (0.3)		
Once or twice	2 (0.2)	2 (0.6)	0 (0)		
3–5 time	1 (0.1)	1 (0.3)	0 (0)		
6–9 times	2 (0.2)	0 (0)	2 (0.3)		
Cannabis				34.21	0.01
Never	918 (93.7)	313 (87.7)	605 (97.1)		
Ever tried	14 (1.4)	6 (1.7)	8 (1.3)		
Once or twice	12 (1.2)	12 (3.4)	0 (0)		
3–5 times	10 (1)	8 (2.2)	2 (0.3)		
6–9 times	9 (0.9)	7 (2)	2 (0.3)		
10 times and more	17 (1.7)	11 (3)	6 (1)		
Hashish				19.14	0.01
Never	949 (96.8)	334 (93.6)	615 (98.7)		
Ever tried	10 (1)	8 (2.2)	2 (0.3)		
Once or twice	4 (0.4)	4 (1.1)	0 (0)		
6–9 times	5 (0.5)	5 (1.4)	0 (0)		
10 times and more	12 (1.2)	6 (1.7)	6 (0.9)		
LSD				1.68	0.19
Never	967 (98.7)	350 (98)	617 (99)		
Ever tried	9 (0.9)	7 (2)	2 (0.3)		
3–5 times	2 (0.2)	0 (0)	2 (0.3)		
10 times and more	2 (0.2)	0 (0)	2 (0.3)		
Heroin				3.73	0.06
Never	973 (99.3)	352 (98.6)	621 (99.7)		
Ever tried	5 (0.5)	3 (0.8)	2 (0.3)		
10 times and more	2 (0.2)	2 (0.6)	0 (0)		
Ketamine				3.68	0.09
Never	971 (99.1)	350 (98)	621 (99.7)		
Ever tried	4 (0.4)	4 (1.1)	0 (0)		
Once or twice	3 (0.3)	1 (0.3)	2 (0.3)		
10 times and more	2 (0.2)	2 (0.6)	0 (0)		

Table S2. *Cont.*

	**Total**	**Males**	**Females**	**KW**	
	**(*N* = 980)**	**(*N* = 357)**	**(*N* = 623)**		
	**f (%)**	**f (%)**	**f (%)**	**H**	***p***
GHB				1.14	0.23
Never	971 (99.1)	352 (98.6)	619 (99.4)		
Ever tried	4 (0.4)	2 (0.6)	2 (0.3)		
Once or twice	4 (0.4)	2 (0.6)	2 (0.3)		
10 times and more	1 (0.1)	1 (0.3)	0 (0)		
Sedatives				8.37	0.04
Never	886 (90.4)	336 (94.1)	550 (88.3)		
Ever tried	63 (6.4)	11 (3.1)	52 (8.3)		
Once or twice	16 (1.6)	2 (0.6)	14 (2.2)		
3–5 times	7 (0.7)	4 (1.1)	3 (0.5)		
6–9 times	2 (0.2)	2 (0.6)	0 (0)		
10 times and more	6 (0.6)	2 (0.6)	4 (0.6)		
Inhalants				0.12	0.73
Never	920 (93.9)	334 (93.6)	586 (94.1)		
Ever tried	34 (3.5)	12 (3.4)	22 (3.5)		
Once or twice	8 (0.8)	2 (0.6)	6 (1)		
3–5 times	2 (0.2)	2 (0.6)	0 (0)		
6–9 times	2 (0.2)	0 (0)	2 (0.3)		
10 times and more	14 (1.4)	7 (2)	7 (1.1)		

© 2016 by the authors; licensee MDPI, Basel, Switzerland. This article is an open access article distributed under the terms and conditions of the Creative Commons Attribution (CC-BY) license (http://creativecommons.org/licenses/by/4.0/).
